# Case Report: Gangrene after one dose of investigational stannic pentetate radiopharmaceutical

**DOI:** 10.3389/fonc.2026.1799473

**Published:** 2026-05-04

**Authors:** Zin W. Myint, Riham H. El Khouli, Bryan P. Lemieux, Charles A. Kunos

**Affiliations:** 1 Division of Medical Oncology, Department of Internal Medicine, University of Kentucky, Lexington, KY, United States; 2 Division of Nuclear Medicine/Molecular Imaging and Radiotheranostics, Department of Radiology, University of Kentucky, Lexington, KY, United States; 3 Radiation Safety, University of Kentucky, Lexington, KY, United States; 4 Department of Radiation Medicine, University of Kentucky, Lexington, KY, United States

**Keywords:** case report, gangrene, radiopharmaceutical, stannic pentetate, tin-117m-DTPA

## Abstract

**Introduction:**

Bone-seeking radiopharmaceuticals are the treatment of choice with clinical benefits for patients with extensive skeletal metastases, but they can also cause serious radiation-related adverse events (rrAEs). Management of rare and severe radiopharmaceutical rrAEs remains challenging because of incomplete knowledge of their natural history and pathogenetic mechanisms. We report a case of unlikely related gangrene following a protracted hospitalization after one dose of investigational [^117m^Sn]tin-117m-diethylenetriaminepentaacetic acid (Sn-117m-DTPA) for treatment of extensive skeletal metastases from castration-resistant prostate cancer (mCRPC).

**Case presentation:**

An 80-year-old man with innumerable painful skeletal metastases from mCRPC developed anorexia and bilateral ankle edema with serous-draining skin fissures of the left foot, worrisome for uninfected dry gangrene, 24 days after receiving a single administration of Sn-117m-DTPA (710 MBq) in a phase II clinical trial (NCT04616547). Laboratory investigations revealed leukopenia (3,180 ml^−1^). Thirteen days later (cycle 1, day 37), he reported anorexia, chills, and generalized weakness, and also had bilateral pedal gangrene. Emergent left above-the-knee amputation and right transphalangeal (ray 2) amputation revealed tissue necrosis complicated by blistering and foul-smelling discharge. He developed postsurgical respiratory decompensation requiring intubation, vasopressors, and broad-spectrum antibiotics. Despite escalating supportive care and enteral feeding, only limited improvement was observed during hospitalization lasting more than 1 month. Five weeks after the onset of pedal gangrene, the patient succumbed to sepsis attributed to a methicillin-sensitive *Staphylococcus aureus* aspiration pneumonia. No autopsy was performed. Remarkably, prior to these events and at sites of skeletal metastases from mCRPC, he reported a substantial reduction in pain (from a score of 7 to 3 on an 11-point scale) and reduced use of oral narcotic analgesia (from three to two times daily).

**Conclusion:**

Gangrene, complicated by leukopenia and sepsis from aspiration pneumonia, is a rarely reported serious adverse event occurring late after a radiopharmaceutical treatment. Given that the window of reversibility for gangrene is narrow, prompt and decisive intervention is warranted. Subtle perturbations in skin integrity after radiopharmaceutical treatment may be more prevalent than realized and should be addressed in both clinical and investigational settings.

## Introduction

More than 80% of men with advanced-stage prostate cancer eventually develop skeletal metastases (1), and these patients often present with debilitating bone pain arising from neuropathic, inflammatory, ischemic, or cancer cell-specific causes (2). Androgen-deprivation therapy by surgical castration or medical suppression remains a primary treatment option (3–7), and treatments that preserve bone health in the context of skeletal metastases often provide palliative benefit (8, 9). Taxane-based chemotherapy also relieves sequelae resulting from tumor-related bone erosion and delays disease progression (10–12). Moreover, a single-fraction stereotactic body radiation therapy (SBRT) treatment may provide short-term relief of cancer-related bone pain (61% at 12 months) for patients with one to three sites of vertebral metastases (13). For those with more extensive skeletal metastases, bone-seeking radiopharmaceuticals such as beta-particle-emitting phosphorous-32, strontium-89, samarium-153, rhenium-186, and lutetium-177 radionuclides, or alpha-particle-emitting radium-223 and actinium-225 radionuclides, have achieved palliation of cancer bone pain but no cure, leaving an unmet medical need (14–20).

[^117m^Sn]tin-117m-diethylenetriaminepentaacetic acid (Sn-117m-DTPA, stannic pentetate) has been developed as a low-energy conversion electron emitter (127, 129, and 152 keV; 13.6-day half-life) with high affinity for bone tissue localization and low soft tissue concentration (21–25). A pilot 15-patient solid tumor skeletal metastases study found durable pain relief without red marrow sequelae after Sn-117m-DTPA infusion (2.6–5.3 MBq kg^−1^) (24). A follow-up phase I/II study that included 30 men with skeletal metastases from prostate cancer also observed bone pain relief (75% at 2+ weeks postdose) without untoward red marrow toxicity after Sn-117m-DTPA infusions (2.6–10.6 MBq kg^−1^) (25). Our single-arm phase II study of Sn-117m-DTPA (10.4 MBq kg^−1^) in men with skeletal metastases from castration-resistant prostate cancer (mCRPC) had as its primary objective the evaluation of sustained pain response (NCT04616547) (26). This case from our phase II study reports clinical decompensation and death from an unlikely related wet gangrene serious adverse event in a man 11 weeks after one dose of the investigational Sn-117m-DTPA radiopharmaceutical.

## Case presentation

An 80-year-old African American man presented with recurrent mCRPC involving several painful (score 7 on an 11-point scale) axial and appendicular skeletal metastases without lymphadenopathy or visceral organ tumors. Primary biopsy-proven hormone-sensitive mCRPC (Gleason 9 = 5 + 4) had been diagnosed 5 years earlier (cT1, cN0, cM1 [grade 5], stage IVB). At initial diagnosis, serologic testing for prostate-specific antigen (PSA) was 1,156 ng ml^−1^, and diagnostic technetium-99 bone scan showed disseminated lesions throughout the spine, pelvic girdle, and bilateral proximal femora. First-line treatment involved bicalutamide, leuprolide, and enzalutamide in a clinical trial (NCT02677896) (27). He achieved a PSA nadir of 1.2 ng ml^−1^. For disease progression, he received leuprolide plus abiraterone/prednisone (or dexamethasone), leuprolide plus cyclophosphamide/prednisone, and off-label pembrolizumab immunotherapy. A liquid biopsy at a prior disease progression revealed a tumor with a low mutational burden and no targetable treatment mutation. His past medical history included stable clinical depression and hypertension complicated by an asymptomatic abdominal aortic aneurysm (3 cm) but without peripheral vascular disease and chronic steroid- and methotrexate-treated symmetrical rheumatoid arthritis of the hands and feet without complicating vasculitis. His activities of daily living were not limited by his comorbidities. Pretrial medications included dronabinol, escitalopram, lisinopril, metoprolol, and oxycodone (5 mg three times daily); these were continued during trial therapy. He quit smoking 40 years earlier. He had no constitutional symptoms such as fever, chills, nausea or emesis, abdominal pain, infection, myalgias, or urinary incontinence. His pretrial Eastern Cooperative Oncology Group (ECOG) performance status was 2, and his screening physical examination revealed a stable, disfiguring bilateral Boutonnière deformity from rheumatoid arthritis. After discussion of various systemic therapeutic options for his mCRPC, he elected to participate in our phase II trial of the Sn-117m-DTPA radiopharmaceutical (NCT04616547) (26). He provided informed consent for treatment and information sharing as part of the research protocol approved by the National Cancer Institute Central and the University of Kentucky Markey Comprehensive Cancer Center Institutional Review Boards. Pretrial PSA was 469 ng ml^−1^; screening chest and abdominopelvic computed tomography scans showed extensive skeletal metastases without lymphadenopathy or visceral organ involvement, and a diagnostic technetium-99 bone scan confirmed axial and proximal appendicular skeletal metastases (**Figure 1**). At baseline, his creatinine was 0.6 mg dl^−1^, and his white blood cell count was 3,580 ml^−1^. He received one dose of intravenous Sn-117m-DTPA (10.4 MBq kg^−1^ or 710 MBq) administered on day 1 (**Figure 2**).

**Figure 1:**
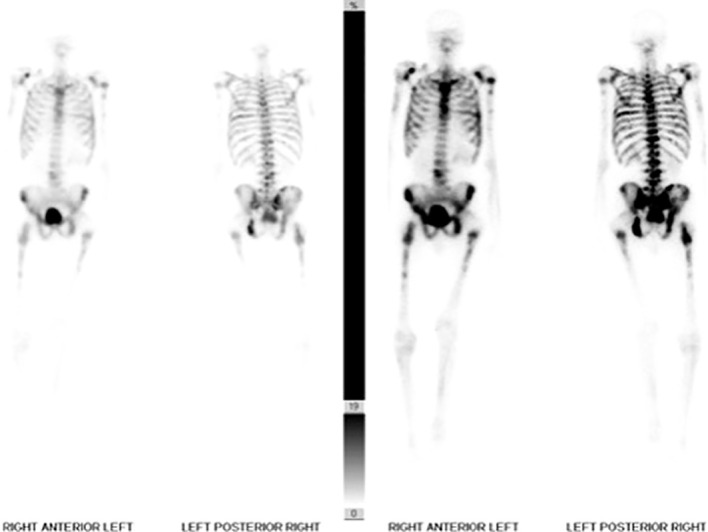
Pretrial screening whole-body planar images taken at 3 h after standard technetium-99m-MDP infusion. Multiple skeletal metastases are present in the axial and proximal appendicular skeleton, with no foci in the bilateral feet. Overlapping intensity involving the bilateral hands and ankles is consistent with rheumatoid arthritis.

**Figure 2:**
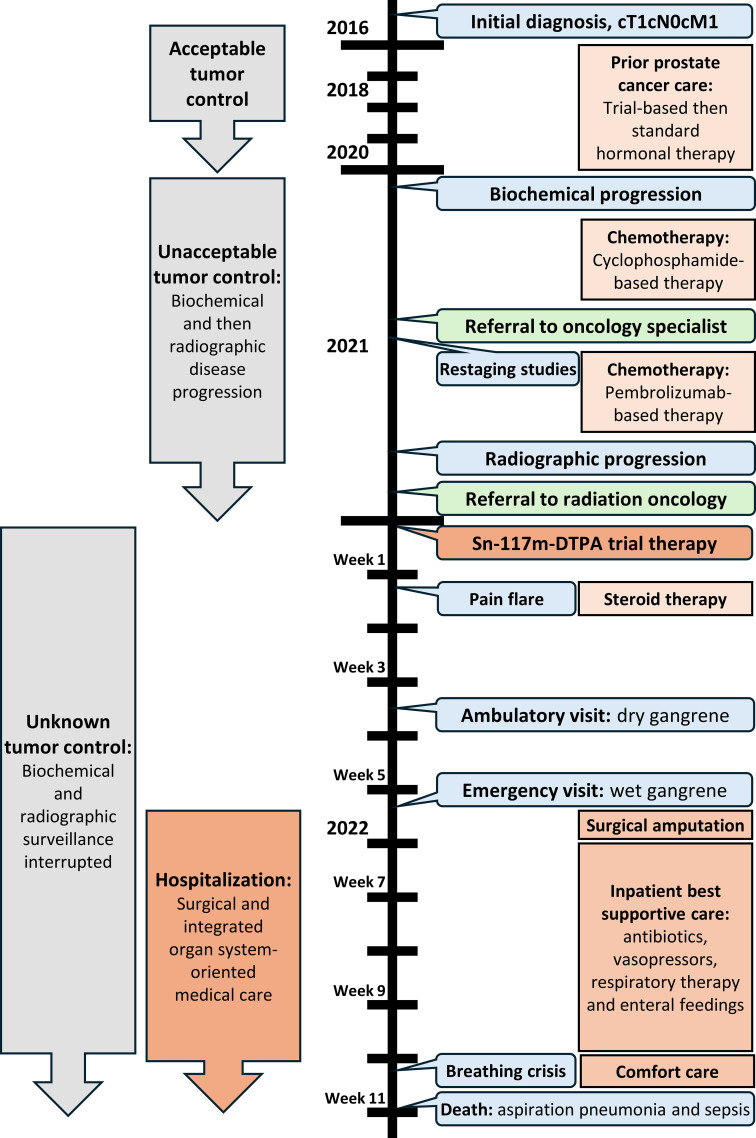
Timeline of pre- and on-trial events. Gray boxes indicate tumor control status. Blue boxes indicate disease-related diagnostic events. Green boxes indicate ambulatory visits. Orange boxes indicate medical interventions or hospital-based care and procedures.

At the time of treatment on day 1, he had no concerning urologic, gastrointestinal, or other constitutional symptoms. Eight days (cycle 1, day 8) after the administration of the first dose of Sn-117m-DTPA, he reported anorexia, nausea that resolved without intervention, and a flare of skeletal bone pain that resolved with a 6-day methylprednisolone dose pack. His white blood cell count was 3,180 ml^−1^ (− 11% from baseline, **Table 1**). Sixteen days later (cycle 1, day 24), he was seen in the clinic and reported improvement in his cancer-related bone pain (score 3 on an 11-point scale) with reduced narcotic use (oxycodone 5 mg two times daily). He also reported persistent anorexia and new bilateral ankle edema with serous-draining skin fissures of the left foot consistent with uninfected dry gangrene. Wound care management was initiated, and ankle-brachial testing was ordered to assess possible peripheral vascular disease. His white blood cell count was now 4,390 ml^−1^ (23% increase from baseline). On day 37 postradiopharmaceutical (cycle 1, day 37), he presented urgently to the hospital afebrile and with complaints of generalized weakness, chills, and wet gangrene involving all five toes and the dorsal surface of the left foot, as well as dry gangrene of the second toe of the right foot. His white blood cell count was only 4,570 ml^−1^ (28% increase from baseline). Broad-spectrum antibiotics were given. Emergent vascular surgery resulted in a left above-the-knee guillotine amputation and a right transphalangeal (ray 2) guillotine amputation. He became apneic after surgery, prompting reintubation and mechanical ventilation. His postoperative chest radiograph showed emphysematous changes without consolidation. Perioperative blood cultures showed no growth. Postoperative ankle-brachial testing of the right leg was unremarkable for peripheral vascular disease waveforms. Aggressive medical management, including a short course of broad-spectrum antibiotics, vasopressors, mechanical ventilation, and enteral feeding, resulted in gradual clinical improvement. Investigations for influenza, coronavirus disease 2019, and respiratory syncytial virus were negative. Additional urine and culture testing for *Streptococcus agalactiae* (group B *Streptococcus*) and *Legionella pneumophila* serogroup 1 were negative. For trial purposes, this clinical presentation of bilateral foot gangrene, resulting in a complex medical course following a single administration of Sn-117m-DTPA, was deemed unlikely to be related to the phase II study agent based on the experience of the study team and an independent data safety monitoring committee (DSMC) review. His leukopenia (or inability to mount a sufficient white blood cell response in the clinical setting of wet gangrene) was deemed probably related to the investigational radiopharmaceutical.

**Table 1:** Patient-specific laboratory trend after a single Sn-117m-DTPA administration.

Biomarker	Baseline	Day 8	Day 24	Day 37	Day 71	Standard	Units
Hemoglobin	12.0	9.9	9.2	8.5	7.2	13.7–17.5	g dl^−1^
Hematocrit	34.8	29.9	28.7	26.0	21.6	40.0–51.0	%
Total leukocyte count	3.58	3.18	4.39	4.57	8.35	3.7–10.3	× 10^9^ L^−1^
Differential leukocyte count							
Neutrophils	61	68	71	77	85	40–80	%
Lymphocytes	25	20	21	17	3	20–40	%
Eosinophils	1	1	0	0	0	1–6	%
Monocytes	10	10	5	3	5	2–10	%
Basophils	0	0	0	0	0	0–1	%
Absolute leukocyte count							
Absolute neutrophils	2.18	2.16	3.11	3.48	7.18	1.60–6.10	× 10^9^ L^−1^
Absolute lymphocytes	0.89	0.63	0.91	0.77	0.25	1.20–3.90	× 10^9^ L^−1^
Absolute eosinophils	0.04	0.02	0.01	0.01	0.00	0.00–0.50	× 10^9^ L^−1^
Absolute monocytes	0.35	0.32	0.22	0.15	0.42	0.30–0.90	× 10^9^ L^−1^
Absolute basophils	0.01	0.01	0.01	0.01	0.00	0.00–0.06	× 10^9^ L^−1^
Platelet count	205	128	121	116	105	155–369	× 10^9^ L^−1^

Evaluating whether this patient suffered from a radiation-related adverse effect (rrAE) of distal extremity gangrene remains challenging because the full-thickness fissures occurred in skin overlying foot bones uninvolved by mCRPC tumors (as evidenced by pretrial technetium-99 bone scan (**Figure 1**) and postoperative surgical pathology), and the skin fissures did not occur from radiopharmaceutical extravasation at the infusion site (since this was in the left arm). In this case, surgical amputation pathology showed mummified toes of the left foot (rays 1 and 2) and black/gangrenous necrotic changes of the left dorsal and plantar foot surfaces without an infectious pathogen. The right second toe (ray 2) showed gangrenous necrosis without an infectious pathogen. Neither pathology specimen had gross or microscopic cancerous tumors identified. Radioactive surveys of the amputation specimens were not performed. The usual cause of radiation-related skin fissures follows a gradual decline in keratinocytes mediated by apoptosis in the more radiation-sensitive epidermal basal layer of the skin. There may be an associated inflammatory reaction resulting from the actions of cytokines and chemokines. These apoptosis-mediated mechanisms do not appear to be present in our case. Serologic tests for interleukin (IL)-1α, IL-1β, tumor necrosis factor (TNF)-α, IL-6, IL-8, C–C motif chemokine ligand 4 (CCL4), C–X–C motif chemokine ligand 10 (CXCL10), and C–C motif chemokine ligand 2 (CCL2) were not performed, as in other clinical scenarios investigating rrAEs (28). The patient did not exhibit any other sites of skin breakdown, skin fissures, or dry/wet gangrene during postradiopharmaceutical monitoring.

Following about 5 weeks after surgical amputation and best supportive care (cycle 1, day 71), his respiratory status decompensated again, and repeat intubation was required. He further declined on the same day, becoming encephalopathic and hypotensive from septic shock attributed to aspiration pneumonia, as determined by his intensive care consultants. Vasopressors and broad-spectrum antibiotics were restarted in the medical intensive care unit. Endotracheal cultures obtained after reintubation grew methicillin-sensitive *Staphylococcus aureus*. Given the absence of substantial improvement over the prior 5 weeks and the consensus of his intensive care consultants, the patient and his family decided to transition to comfort care. He underwent palliative extubation. Comfort care continued for another 6 days. On day 77 postradiopharmaceutical (cycle 1, day 77), the patient died from progressive aspiration pneumonia and sepsis complicated by multiorgan failure. No autopsy was done. No imaging tests were performed to investigate the pathogenetic response of mCRPC to a single dose of the Sn-117m-DTPA radiopharmaceutical. One serum PSA level was obtained during his postradiopharmaceutical hospitalization and was 539 ng ml^−1^ (15% increase from baseline).

## Discussion and conclusions

Radiopharmaceuticals improve cancer outcomes but can be associated with variable rrAEs. In the case described above, a single dose of the bone-seeking Sn-117m-DTPA radiopharmaceutical led to an improvement in skeletal pain from mCRPC and a reduction in analgesic narcotic use but was unfortunately complicated by an unlikely related bilateral pedal gangrene requiring hospitalization and surgical amputation, which ultimately proved fatal due to progressive aspiration pneumonia and sepsis after a complicated and protracted clinical course.

The striking clinical findings in this case were leukopenia and a resulting lack of immune response to progressive pedal wet gangrene and necrotic tissue. Adverse red marrow toxicity began about 3 weeks after irradiation and a little more than 2 weeks after high-dose methylprednisolone was given to palliate a flare in skeletal bone pain. Both radiation and glucocorticoid exposures have been associated with immunosuppression and vascular injury (25, 29–32). For Sn-117m-DTPA in particular, there is deposition of the agent in actively mineralizing cancellous bone osteoid interfaces within red marrow (22), rendering leukopenia or anemia a probable rrAE after each administration. When confounded by leukocyte dysfunction from glucocorticoid exposure (31, 32) and possibly prior chronic steroid and methotrexate exposure for treatment of his rheumatoid arthritis, an insufficient immune response to gangrene and necrotic tissue becomes explainable. Large-field external beam radiation therapy (i.e., half-body irradiation [HBI]) (33) or taxane-based chemotherapy (34) could potentially exacerbate these effects as well, but did not apply in our case, as the patient did not undergo these therapies. Our current case shares rapid-onset and prolonged leukopenia similar to that observed in two other clinical cases of this agent (**Table 2**). In a pilot study, a man with metastatic prostate cancer had his white blood cell count fall 7 days after a single dose of Sn-117m-DTPA (388 MBq), and the counts remained low for the next 10 weeks (24). In a phase I/II trial, three women with metastatic breast cancer had reduced white blood cell counts between 7 and 21 days after single doses of Sn-117m-DTPA (296, 311, and 703 MBq each) (25). As neither of these reports evaluated the red marrow radiation-absorbed dose prior to these hematologic assessments, cause-and-effect relationships cannot be established. Our case also does not permit this association, supporting a formal study objective of red marrow radiation dosimetry in future clinical trials of this agent.

**Table 2:** Adverse events for Sn-117m-DTPA (24, 25)^*^.

Event	Sn-117m-DTPA (N = 55)			
	All Grades	Grade >3		
	*number of patients (percent)*			
Any adverse event	13 (24)	1 (2)		
Leukopenia (x10^9^ L^-1^)	12 (2)	1 (2)		
Adverse event that led to reduction in Sn-117m-DTPA dose	0 (0)	0 (0)		
Adverse event that led to interruption of Sn-117m-DTPA	1 (2)	0 (0)		
Adverse event that led to discontinuation of Sn-117m-DTPA	0 (0)	0 (0)		
1998 National Institutes of Health criteria†				
	1 (mild)	2 (moderate)	3 (severe)	4 (unacceptable)
Leukopenia (x10^9^ L^-1^), reference	3.0—3.9	2.0—2.9	1.0—1.9	<1.0
Leukopenia (x10^9^ L^-1^), number of patients (percent)	8 (15)	3 (5)	1 (2)	0 (0)

*Adverse events during treatment were those that occurred on or after the start of agent infusion and up to 30 days after the last administration of the agent or before subsequent anticancer treatment.

†Trials reported leukopenia according to the indicated adverse event criteria.

The full spectrum of leukopenia from bone-seeking radiopharmaceuticals such as Sn-117m-DTPA remains undetermined but is likely broad and heterogeneous among patients. Other investigators have reported red marrow toxicity from beta- and alpha-particle-emitting radionuclides (14–20), but correlating radiation-absorbed dose and timing of white blood cell count nadir remains a speculative clinical observation (typically occurring 2 to 4 weeks after radiopharmaceutical administration). Whether leukopenia in our patient contributed to the progression of bilateral pedal gangrene remains speculative, but the protracted failure of his immune cells to mount an adequate response appears likely attributable to the radiopharmaceutical.

Determining a differential diagnosis for gangrene remains an important clinical step in establishing temporal associations of symptoms and signs of disease. Advanced age, male sex, and frailty elevate the risk for peripheral vascular disease and amputation (35). Our patient fits each of these descriptors, all of which are contributory to his eventual medical course and amputations. Immunosuppressant drugs like glucocorticoids or methotrexate do not increase the risk of extremity amputation (36); his prior or concomitant use of these agents is therefore unlikely to be causative in this instance. Both hypertension and smoking increase the likelihood of peripheral vascular disease, gangrene, and amputation (37). Despite no symptoms of claudication in our patient, his preexisting hypertension likely conditions his extremities to a higher risk of limb or distal pedal amputation. A long period of smoking cessation (such as his 40-year abstinence) lowers long-term amputation risk (38). A hypercoagulable state of malignancy is associated with activation of the coagulation cascade and disruption of fibrinolytic pathways (39), and if microvascular ischemia impairs limb perfusion, limb viability becomes threatened (40). Mid-sized artery rheumatoid vasculitis can also be implicated (41). In our case, there was no sonographic or pathologic evidence of acute limb vascular occlusion or vasculitis. Lastly, no pathogens were detected that would result in infection-related gangrene.

The conspicuous full-thickness skin fissures of the feet requiring eventual surgical amputation require scrutiny of the radiopharmaceutical as a potential triggering agent. To our knowledge, this is the first case in which a therapeutic radiopharmaceutical and gangrene occurred within a single medical course. At first consideration, the progression from no skin toxicity to serous fluid-draining skin fissures to wet gangrene over a 5-week timeframe raises the question of whether the radiopharmaceutical should be considered contributory. The gangrene occurred away from the infusion site (thereby excluding radionecrosis from agent extravasation) and not at a site of tumor involvement (thereby excluding high agent deposition at a bone–tumor interface as a cause), and it was determined by an independent DSMC to be unlikely related to the investigational agent. However, the focal nature of the pedal skin fissures at onset suggests another possible explanation for this radiopharmaceutical’s involvement. Radioactive [^117m^Sn]tin in its microparticle formulation is used to treat canine and human inflammatory arthritides. Radiosynoviorthesis is a minimally invasive procedure in which radionuclides such as [^117m^Sn]tin in microparticle formulation are injected directly into the inflamed synovial lining of a joint (42). The procedure has been studied in canines (43, 44), and a human phase I clinical trial of the radioactive microparticle has been considered in Canada (CTIN228513). There have been no reports of wet gangrene in canines after direct injection (43); whether a systemic injection of Sn-117m-DTPA might colocalize in phalangeal joints of a patient with extensive rheumatoid arthritis, as possibly occurred in our patient, remains uncertain. Rheumatoid arthritis-inflamed synovium (pannus) induces bone destruction (45), promoting bone remodeling and calcium exchange, both of which might facilitate Sn-117m-DTPA localization. Unfortunately, due to a lack of a radioactive survey of our patient’s amputated tissues, this case does not provide evidence to confirm or refute this hypothesis. We urge further investigation of this putative phenomenon as an adverse event of special interest in clinical trials of the Sn-117m-DTPA radiopharmaceutical.

The rarity of reported skin fissures complicated by wet gangrene as a potential rrAE highlights the need to discuss cases such as this so that early recognition of changes in skin integrity might prompt intervention. While skin breakdown is frequently reported as an adverse reaction in trials of external beam radiotherapy, this rrAE has not typically been studied in trials of radiopharmaceuticals. It is unclear whether cutaneous or subcutaneous tumor involvement contributes to radiation-related desquamation or ulceration with a mild presentation, or whether this represents a rare phenomenon that tends to be severe when off-target tissue antigens, receptor proteins, or specific binding partners are expressed. We suggest close monitoring of skin integrity changes in clinical and investigative settings and maintaining a high index of suspicion for toxicity, as there is potential for progressive, severe, and likely irreversible injury if disrupted skin integrity is left unchecked.

## Data Availability

The raw data supporting the conclusions of this article will be made available by the authors, without undue reservation.
